# Environmental Drivers Controlling Bacterial and Archaeal Abundance in the Sediments of a Mediterranean Lagoon Ecosystem

**DOI:** 10.1007/s00284-018-1503-3

**Published:** 2018-05-15

**Authors:** Claudia Pala, Massimiliano Molari, Daniele Nizzoli, Marco Bartoli, Pierluigi Viaroli, Elena Manini

**Affiliations:** 1 Department of Chemistry, Life Sciences and Environmental Sustainability, University of Parma, Parma, Italy; 2 Institute for Marine Science - ISMAR, National Research Council of Italy – CNR, Ancona, Italy; 3 Max Planck Institute for Marine Microbiology, Celsiusstrasse 1, 28359 Bremen, Germany

## Abstract

The environmental factors controlling the abundance of *Bacteria* and *Archaea* in lagoon ecosystems are poorly understood. Here, an integrated physico-chemical, biogeochemical, and microbiological survey was applied in the Sacca di Goro lagoon (Po River Delta, Italy) to investigate the variation of bacterial and archaeal abundance, as assessed by Fluorescence In Situ Hybridization, along winter and summer environmental gradients. We hypothesised that bacterial and archaeal cells respond differentially to physico-chemical parameters of the sediment, which can be manifested in variations of total cells number. Our results suggest that *Archaea* are an important component of microbial communities (up to 20%) and they are also quite constant along the sediment depth investigated, while *Bacteria* tend to decrease in the subsurface sediments. The abiotic (i.e. temperature, ammonium, pH) and trophic parameters (i.e. chlorophyll *a*) explain differentially the variations of bacterial and archaeal distribution, and raise interesting questions about the ecological significance of the microbial composition in this area.

**Electronic supplementary material:**

The online version of this article (10.1007/s00284-018-1503-3) contains supplementary material, which is available to authorized users.

## Introduction

The distribution of *Bacteria* in marine sediments have been extensively investigated [e.g. 9, 13, 19, 31, 33, 44, 57] and a variety of environmental factors have been reported to influence their abundance, such as sediment depth [20], salinity [47], phosphorus [27], organic matter [26], and pH [23]. Only recently, the role of *Archaea* in biogeochemical processes started to be elucidated, showing that they are involved in organic matter transformation such as methanogenesis [18], anaerobic methane oxidation (AMO, [8]), protein degradation [25], and aerobic ammonium oxidation [22]. Thus, only few studies quantified the abundance of *Archaea* in marine sediments, and investigated their variation with changes of environmental settings. These studies identified factors such as sediment depth, salinity, temperature, and food supply as the main archaeal environmental constrainers [15, 16, 20, 30].

Even less is known about the distribution of benthic *Bacteria* and *Archaea* in response to changes of environmental settings in aquatic transitional ecosystems, such as lagoons. Transitional ecosystems are generally characterised by strong physico-chemical gradients (i.e. temperature, salinity, nitrogen, pH, and organic matter), which make these systems highly unstable and often subject them to unpredictable fluctuating conditions [51, 53]. They typically behave as sinks for organic carbon [39], and the export of accumulated detritus towards higher trophic levels is mainly mediated by the functioning of the benthic microbial loop [26]. In these ecosystems, microbes control the flux of C and N to the atmosphere and within the trophic webs [5]. For a better understanding of the functioning of lagoons, and how human pressure can impact this ecosystem, it is important to clarify how environmental factors can shape microbial communities.

**Fig. 1:**
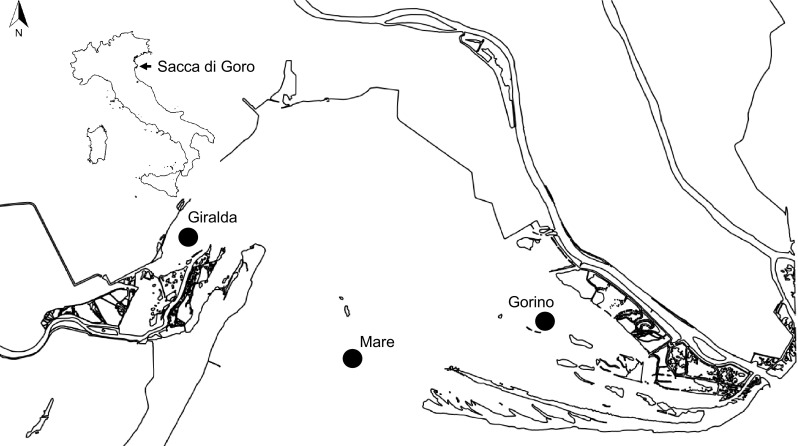
The sampling area, Sacca di Goro Lagoon (44.78–44.83°N, 2.25–12.33°E), and the three sampling sites Giralda, Gorino, and Mare

The Sacca di Goro (Italy) is a Mediterranean lagoon (Fig. 1) located in the southern part of the Po River Delta. As common feature of coastal lagoons, the Sacca di Goro is a transitional area characterised by shallow waters, reduced hydrodynamics, high productivity, and high sedimentation rates [53]. It is, therefore, sensitive to the phenomena of evaporation and precipitation, which, together with tidal currents, cause rapid changes in the chemical and physical properties, i.e. strong fluctuations in oxygen and sulphide concentrations [51]. The lagoon is also subjected to high anthropogenic pressures, receiving inputs of nutrients derived from organic and inorganic effluents of urban sewage. Furthermore, the Sacca di Goro is intensively exploited through farming of bivalves. All these factors alter the natural processes of the ecosystem causing eutrophication and dystrophy [54]. For this reason, this site is an ideal, natural laboratory to investigate the effects of strong environmental changes on bacterial and archaeal abundance and distribution. This lagoon has been well characterised from a biogeochemical point of view [51–54], but so far only few works have studied its microbiology. Manini et al. in 2003 [26] analysed the total microbial community, while Danovaro and Pusceddu in 2007 [13] provided information about bacterial and archaeal community structure using FISH and bacterial composition using ARISA. Both studies focused on the surficial sediments and only in one station of Sacca di Goro.

In this work, we have quantified the abundance of benthic *Bacteria* and *Archaea* along spatial and temporal environmental gradients of the Sacca di Goro lagoon via Fluorescence In Situ Hybridization. Alongside, we determined several environmental parameters to correlate with the observed cells number, aiming to identify community drivers. We found that differences in bacterial and archaeal abundance are correlated with physico-chemical parameters of the sediment and water/sediment interface. Additionally, we compared surface and subsurface sediment communities in terms of *Bacteria* versus *Archaea* dominance, and revealed a shift in microbial community composition with sediment depth towards higher archaeal abundance.

## Materials and Methods

### Site and Sampling Description

The study was carried out in the Sacca di Goro (Fig. 1), a semi-enclosed coastal lagoon of approximately triangular shape with a surface area of 26 km^2^ and an average depth of 1.5 m. Samplings were carried out during two distinct periods of the year: spring/summer (June 2011) and winter (February 2012) using a hand-operated motorboat. We chose three sampling sites in the lagoon named, Giralda, Gorino, and Mare (Fig. 1). They were selected according to pre-determined concentration of ammonium, dissolved oxygen, salinity, temperature, and pH, i.e. representing sites along environmental gradients [52]. The station Giralda is characterised by silt–clay sediment, low salinity, and low hydrodynamics. Gorino is characterised by muddy sediment, and the presence of macroalgae during the summer that often caused anoxia and a local dystrophic crisis. Mare is closest to the Adriatic Sea and is thus characterised by sandy sediment and high salinity. At each site, sediment cores (transparent plexiglas liners of 8 cm diameter and 30 cm height) were collected in triplicates. The sediment was sliced into three different layers (0–0.5 cm, 0.5–2 cm, 2–5 cm) and kept separately at 4 °C until analysis.

The overlying water and upper layers of the sediment were tested for physico-chemical parameters. The overlying water (100 ml) was immediately filtered (0.7 µm GF/F) and stored at 4 °C until analysis. Within 24 h after collection, water samples were analysed for ammonium, nitrite, and nitrate concentrations.

### Environmental Parameters

Salinity, temperature, pH, dissolved oxygen, and redox potential (Eh) were measured in situ using a multiparametric probe (YSI Instruments, Yellow Springs, OH USA, model 556). Data points were collected from the overlying water in the core down to ca. 5 cm sediment depth. Ammonium, nitrite, and nitrate were determined in the overlying water.

Ammonium was determined spectrophotometrically using salicylate and hypochlorite in the presence of sodium nitroprusside [10]. Nitrite was determined spectrophotometrically after diazotisation reaction. Nitrate was determined spectrophotometrically after cadmium reduction of nitrate to nitrite [4], followed by the nitrite determination protocol.

### Biochemical Composition of the Sedimentary Organic Matter

Total protein (PRT), carbohydrate (CHO), lipid (LIP), and chlorophyll *a* (CHLA) were determined according to Danovaro [14]. Concentrations were calculated using standard curves and normalised to sediment dry weight after desiccation (60 °C, 24 h). Protein, carbohydrate, and lipid concentrations were converted into C equivalents using the conversion factors of 0.49, 0.40, and 0.75 µg C µg^−1^, respectively [17]. Biopolymeric organic C (BPC) was calculated as the sum of the C equivalents of protein, carbohydrate, and lipid, and this was used as a proxy for the available trophic resources [40].

### Fluorescence In Situ Hybridization (FISH) and Total Microbial Cells Number

The microbial community structure was investigated using Fluorescence In Situ Hybridisation (FISH) [3]. Oligonucleotide probes labelled with a Cy3 fluorochrome at the 5′ end were purchased from Biomers (Biomers.net, Germany). We used universal probes for *Bacteria* (EUB338: 5′-GCTGCCTCCCGTAGGAGT-3′ [2]; EUB338II: 5′-GCAGCCACCCGTAGGTGT-3′ [12]; EUB338III: 5′-GTCGCCACCCGTAGGTGT-3′ [12]), for *Archaea* (Archaea915: 5′-GTGCTCCCCCGCCAATTCCT-3′ [46]), and a negative control probe for non-specific binding (NONEUB: 5′-ACTCCTACGGGAGGCAGC-3′ [55]) with 35% of formamide concentration in the hybridisation buffer. Hybridisations and microscopy counts of hybridised and DAPI (4,6-diamidino-2-phenylindole)-stained cells were performed as previously described [37, 38], (see Supplementary Materials for details).

The counting was performed with an epifluorescence microscope (Zeiss Axioskop 2) and the following filter sets: DAPI: Ex 359 nm and Em 441 nm: Cy3: Ex 546 nm and Em 575 nm. Randomly, 20 fields of view were counted over the entire area of the filter for a total number of cells higher than 700 per filter piece.

### Statistical Analysis

Principal component analysis (PCA) was performed to highlight differences in environmental settings among different sites, layers, and sampling time. Normality and homoscedasticity of data were examined, and, when required, data were transformed (e.g. *Z* scoring, square root) and newly tested. The spatial variability of the bacterial and archaeal abundance and relative abundance, expressed as a percentage of total cells, were investigated using univariate analysis of variance (ANOVA). Tukey post hoc comparison tests (*α* = 0.05) were applied when significant differences were encountered to identify the presence of patterns. In case the assumptions for an ANOVA were not met, Kruskal–Wallis test and post hoc Dunn test were performed. For testing the effect of space (“Site” and sediment “Layer”) and time (“Season”) on variations observed in bacterial and archaeal abundance, multifactorial ANOVA analyses were performed based on a three factors design with Layer nested in Site. Linear regression (LR) analysis was performed to investigate which environmental variable or set of variables could better explain the patterns in bacterial and archaeal abundance. Prior to the analyses, the explanatory environmental variables were transformed (i.e. *Z* scoring) and diagnosed for collinearity based on the variance inflation factor (VIF). Only variables with VIF < 5 were used in regression analysis (i.e. CHLA, BPC, pH, T, ammonium). Additionally, variation partitioning (VP) allowed to identify how much of the variation in bacterial and archaeal abundance was explained by the significant contribution of the explanatory variables returned by LR. All statistical analyses were performed in R (version 3.3.0 [41]) using packages vegan [36], usdm [34], and ggplots2 [56]. All results are represented in average values with standard deviation (±).

## Results

### Physico-chemical Parameters

Mare located closest to the Adriatic sea, had the highest salinity values (25‰ in summer and 29‰ winter), temperature was lower in summer (23 °C) and higher in winter (13 °C), and the redox potential (Eh) value was the most positive (+398 mV) of all stations during winter. The pH value was 7.8. Dissolved oxygen (DO) did not show significant differences among the sampling stations, but was generally higher in winter than in summer (Tables 1, 2). Giralda is located closest to the river mouth and had the lowest salinity (5‰). The highest and lowest values for water temperature were also recorded here, with 27.8 °C in summer and 7.8 °C in winter. Among the three stations Giralda showed highest nitrate, nitrite, and ammonium concentrations in winter (125, 5, and 80 µM, respectively) and in summer (48, 3, and 48 µM, respectively). At Giralda, the lowest pH values were measured, 7.2 during summer. At all other stations pH values were higher, especially during the winter, with highest pH value (8.3) recorded at Gorino (Tables 1, 2). Gorino, as the intermediate station between the Po river delta and the sea, showed intermediate concentrations of nitrate, nitrite, and ammonium during summer (2.2, 0.6, and 9 µM, respectively) and winter (30, 1, and 15 µM, respectively; Table 1).

**Table 1:** Physico-chemical parameters in the overlaying water of Mare, Gorino, and Giralda during summer and winter

Season	Station	Depth (m)	*T* (°C)	Salinity (‰)	DO (%)	Ammonium (μM)	Nitrite (μM)	Nitrate (μM)
Summer	Mare	1.5	23.3	25	82	12	3	7
	Gorino	0.9	27.3	18.6	80	9	0.6	2.2
	Giralda	0.5	27.8	5	70	48	3	48
Winter	Mare	1.5	13	29	127	7.5	2.9	192
	Gorino	0.9	8	24	89	15	1	30
	Giralda	0.5	7.8	9	94	80	5	125

**Table 2:** pH and Eh measurements in the three sediment layers of Mare, Gorino, and Giralda during summer and winter

Season	Station	Layer	pH	Eh (mV)
Summer	Mare	1	7.8	205.3
		3	7.9	195.3
	Gorino	1	8.2	106.4
		2	7.5	−80.1
		3	7.6	−172.8
	Giralda	1	7.5	94.3
		2	7.3	−172.4
		3	7.2	−179.9
Winter	Mare	1	7.5	398
		2	nd	nd
		3	nd	nd
	Gorino	1	8.3	107
		2	8	22
		3	7.9	−248
	Giralda	1	7.8	198
		2	7.7	182
		3	7.7	121

Layer 1 = 0–0.5 cm, layer 2 = 0.5–2 cm, layer 3 = 2–5 cm

*nd* not detected

The redox potential (Eh) at Giralda and Gorino was determined down to 5 cm of the sediment. During summer, Eh values were positive at the surface (+94.3 for Giralda, and +106.4 for Gorino). At layer 2 (0.5–2 cm), the sediment became chemically reduced with Eh values of −172.4, and −80.1 mV. Layer 3 (2–5 cm) had the most reduced conditions (−179.9, and −172.8). In winter, the Eh values in the deeper parts of the sediment stayed positive at Giralda (+121 at layer 3), but were strongly reduced at Gorino (−248 at layer 3; Table 2).

### Organic Matter Composition

At Giralda, the sediment surface contained more organic matter than the deeper layers, both in summer and winter (10.0 ± 1.1 mg C g^−1^ at the surface, and 7.2 ± 0.9 mg C g^−1^ in layer 3). At Gorino, this trend was reversed, especially in winter, ranging from 5.6 ± 0.6 mg C g^−1^ at the surface to 10.8 ± 1.4 mg C g^−1^ at layer 3. Mare showed the lowest values of organic matter among the three stations, but the surface layer was again more enriched in organic matter than the deeper layers, especially in winter, ranging from 1.6 ± 0.3 mg C g^−1^ at the surface to 0.5 ± 0.02 mg C g^−1^ at layer 3, Fig. S1. The analysis of the composition of sedimentary organic matter (OM) showed that the dominant fraction was composed of total protein (PRT), on average 53%. Furthermore, lipids (LIP) made up 26% and carbohydrates (CHO) 22% of the total organic matter. Biopolymeric carbon (BPC) was highest at Giralda, both in summer and winter, and lowest at Mare (Fig. S1).

Chlorophyll *a* (CHLA) concentrations were highest at the surface sediment of Gorino in winter (8.2 ± 2.4 µg g^−1^). Both at Gorino and Giralda, the chlorophyll *a* was higher in winter than in summer. Mare had the lowest chlorophyll *a* concentrations compared to the other stations, with higher values of 0.6 ± 0.1 µg g^−1^ at the surface in summer than winter. In all stations, chlorophyll *a* concentrations decreased with sediment depth (Fig. S1).

### Bacterial and Archaeal Abundance

**Fig. 2:**
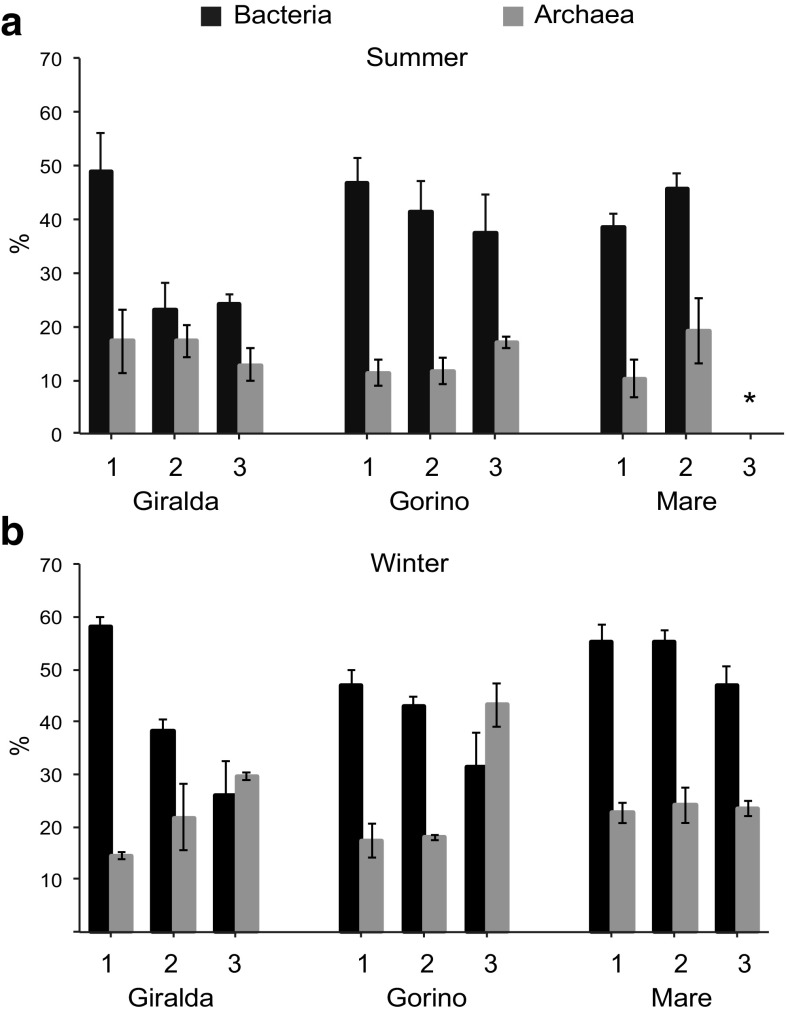
Bacterial and archaeal relative abundance at Giralda, Gorino, and Mare during **a** summer 2011, asterisk represents not detected, and **b** winter 2012. The numbers 1, 2, and 3 indicate the different sediment layers. Layer 1 (0–0.5 cm), layer 2 (0.5–2 cm), and layer 3 (2–5 cm)

**Fig. 3:**
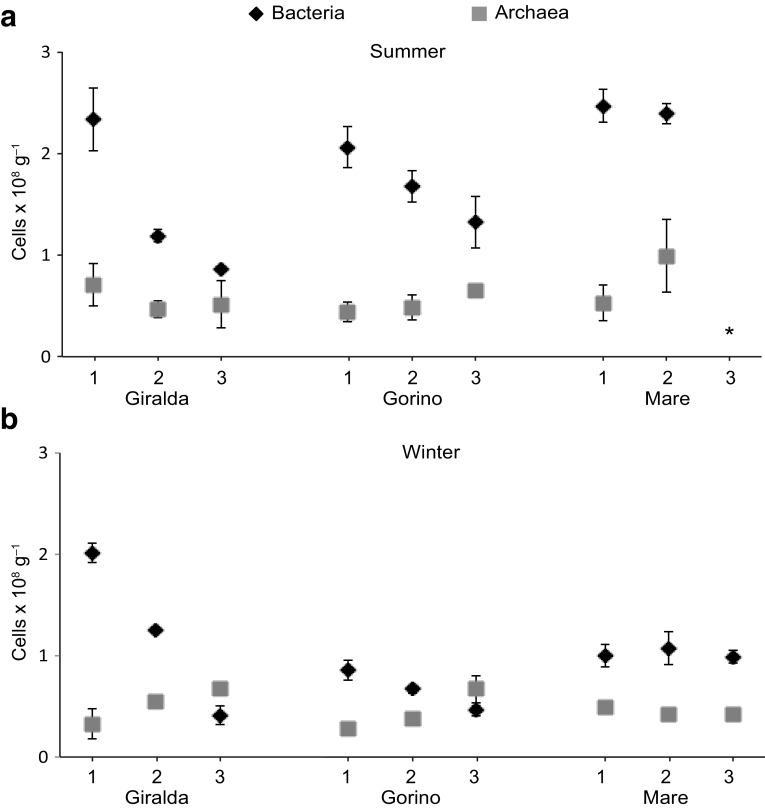
Bacterial and archaeal abundance at Giralda, Gorino, and Mare during **a** summer 2011, asterisk represents not detected, and **b** winter 2012

In this study, we applied Fluorescence In Situ Hybridization (FISH) to enumerate the abundance of *Bacteria* and *Archaea* cells in sediments of the Sacca di Goro lagoon. On average, the sum of bacterial and archaeal abundance relative to DAPI-stained cells ranged between 51 ± 8% and 70 ± 10% in summer and winter, respectively, (Fig. 2a, b). Bacterial and archaeal cells number were on average higher in summer than in winter (ANOVA, *P* < 0.001). During both seasons, *Bacteria* were always higher in abundance than *Archaea*, with bacterial cells number usually peaking at the sediment surface and decreasing with sediment depth (ANOVA, Tukey test, *P* < 0.01). *Archaea* showed an opposite trend with higher abundance in deeper sediment layers (ANOVA, Tukey test, *P* < 0.01; Fig. 3a, b). At Giralda, both, in summer and winter, bacterial cells number strongly decreased with sediment depth (from ca. 2 to ca. 0.5 cells × 10^8^ g^−1^), while *Archaea* remained at 0.5 cells × 10^8^ g^−1^ in all layers during the summer, but increased slightly with depth in winter (0.3–0.7 cells × 10^8^ g^−1^). At Gorino, the opposing downwards trends of *Archaea* and *Bacteria* can be observed for both seasons, although bacterial cells number are lower during winter (decreasing from 1.7 cells × 10^8^ g^−1^ to 0.7 cells × 10^8^ g^−1^). At Mare, bacterial cells number were especially high in summer (highest numbers across all stations; 2.5 cells × 10^8^ g^−1^), but without a clear downwards trend, Fig. 3a, b. The *Archaea* during summer at Mare showed again the trend of increasing numbers with depth, reaching highest numbers for all stations and seasons (1.0 cells × 10^8^ g^−1^). In winter, bacterial cells number at Mare decreased substantially (to ca. 1.0 cells × 10^8^ g^−1^) and again showed no depth trend. Also statistically, the bacterial abundance at Mare was significantly higher than at Gorino and Giralda in summer (ANOVA, *P* < 0.05), and at Gorino in winter (ANOVA, *P* < 0.001), (Fig. 3a, b).

## Discussion

The transition from the continental to the marine domains determines steep gradients of the environmental parameters that, from one hand promote unique ecosystems services, and on the other side make this habitat the most vulnerable coastal system to climatic and human pressure. Sacca di Goro is recognised as an experimental field site for studying Mediterranean watershed–coastal lagoon interactions, where microbes play a major role in organic matter remineralisation and in carbon transfer to higher trophic levels [26]. However, the lack of information about microbial abundance, community composition, and diversity are the major gaps for understanding the coastal lagoon biogeochemical cycling and the role of microbes in buffering the increasing anthropogenic impact. In this study, for the first time, we described the temporal and spatial variation of bacterial and archaeal abundance in the Sacca di Goro lagoon sediments. Specifically we investigated the effects of seasonality and physico-chemical gradients, typically reported for this area [52] and here confirmed, on the abundance of *Bacteria* and *Archaea* in order to characterise the microbial baseline standing stock variability.

The FISH recovery efficiency observed here is consistent with previous reports for marine benthic habitats [31]; however, we found a larger contribution of *Archaea* to the microbial community (on average 20 ± 3%; Fig. 2a, b), which is typically <10% in other coastal marine sediments [20, 31]. Sediment depth was an important factor controlling microbial community structure, especially for the internal stations of Sacca di Goro lagoon (Table S1). The contribution of *Archaea* to the total microbial community tended to be constant or increased with sediment depth, especially during winter. The relative abundance of *Bacteria* decreased with depth during both seasons (Fig. 2a, b). This pattern is not unusual for marine sediments; however, the shift from *Bacteria*-dominated to *Archaea*-dominated microbial communities has been shown to occur at sediment depth >50 cm in coastal habitats [31]. In anaerobic sediments, *Archaea* play a crucial role in methanogenesis [18], methane oxidation [8], and the degradation of detrital protein [25]. Archaeal methanogens (e.g. *Methanomicrobia* and *Methanobacteria*) are typically described in anoxic marine sediments rich in organic matter [48], and *Archaea* responsible for anaerobic methane oxidation can constitute up to 28% of total microbial community in the Baltic Sea sediments at 25 cm depth [49]. The *Marine Benthic Group B (MBGB)* and *Miscellaneous Crenarchaeotal Groups (MCG*) are other important archaeal taxa described in anoxic sediments of aquatic transitional habitats [24], which play an important role in anaerobic organic matter remineralisation [45].

The high productivity, low hydrodynamics, and presence of cohesive sediments (alluvial mud with high clay and silt contents) produced accumulation of organic matters at Giralda and Gorino, as a common feature of Mediterranean coastal eutrophic lagoons [13, 26, 39]. The presence of cohesive sediments and low hydrodynamics contributes keeping the sediment layers quite stable and low oxygenated [58]. Under these conditions, the high content of organic matter at Giralda and Gorino stimulates mineralisation rates [26], which in turn are responsible for quick oxygen consumption in the upper millimetres of sediment and the production of reduced compounds (e.g. H_2_S and CH_4_) in subsurface sediments [42, 59 M. Bartoli personal communication]. We hypothesised that the high OM degradation rates and low hydrodynamics generated a sharp redox cline in the first top centimetres of Gorino and Giralda sediments, which may have raised the sulphate–methane-transition zone [29], then favouring archaeal communities typically described for deeper anaerobic sediment layers [8, 18, 25]. In this scenario, our findings suggest that in anoxic sediments of highly productive coastal lagoons, such as Sacca di Goro, *Archaea* could play a so far neglected role in carbon cycling.

**Fig. 4:**
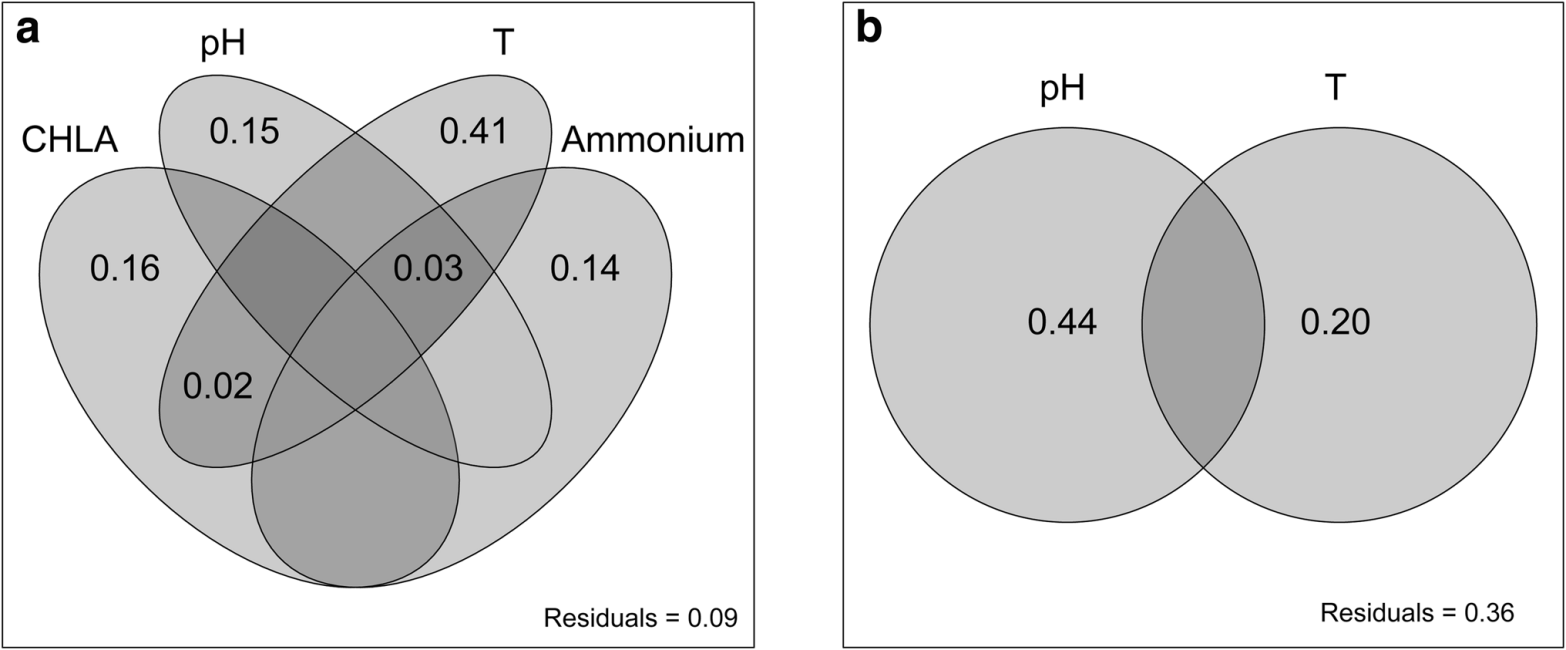
Variation partitioning of variables explaining variance of **a** bacterial and **b** archaeal abundance

The seasonality was a principal source of variability of bacterial and archaeal standing stocks (Table S1). Focussing on the data from the surface sediment layer, the variables strongly affected by seasonality and riverine incoming waters (i.e. temperature, CHLA, pH, ammonium) explained a large proportion of variance of bacterial and archaeal abundance (Fig. 4a, b). In particular, the abundance of *Bacteria* was mainly driven by temperature, and equally strong by CHLA, pH, and ammonium (Fig. 4a). Conversely, the abundance of *Archaea* was mainly driven by pH, and secondary by temperature (Fig. 4b).

Biological activity (i.e. metabolic and chemical reaction rates) increases exponentially with temperature [11], and thus availability of thermal kinetic energy is one of the main controlling factors of benthic microbial assemblages [28]. The low temperature of lagoon water in winter reduces the microbial activity, which likely resulted in lower bacterial density. Little variation of *Archaea* abundance in surficial sediments observed between summer and winter suggests that they are more tolerant than *Bacteria* to temperature decrease. Thermal adaptation seems to be a common feature among *Archaea*, as they can be abundant in different low-temperature environments [15, 21, 32, 50].

Organic carbon availability (i.e. chemical potential energy) in marine sediments is recognised as another important factor that affects the metabolism, distribution, and dynamics of benthic microorganisms [7, 15, 19]. Specifically, under trophic resource-limiting conditions, heterotrophic *Bacteria* and *Archaea* are primarily controlled by BPC and POC-flux [7, 15]. However, we could not identify a clear relationship between the amount and composition of OM and bacterial abundance. Lagoon sediments have a generally high load of organic matter in, which therefore might not have acted as a limiting factor for Sacca di Goro microbial communities. The quality, i.e. freshness of OM as measured in CHLA had an important role in controlling the bacterial abundance (Fig. S2a). Chlorophyll *a* is used as a proxy of freshness because it represents the fraction of photosynthetically produced organic matter that has not been degraded, yet [40]. The fact that *Archaea* are not affected by the availability and composition of organic matter in superficial sediments suggests that they can use different energy source. Mostly of the *Archaea* found in aerobic aquatic environments are ammonium oxidisers [35], autotrophs using ammonium as electron donor and oxygen as electron acceptor in the respiratory chain. The distribution of bacterial and archaeal ammonium oxidisers in estuarine environments has been shown to depend of salinity [43], oxygen concentration [47], CHLA [1], and temperature [6]. Erguder et al. in 2009 [16] proposed specific niches for bacterial and archaeal ammonium oxidisers corresponding to varying dissolved oxygen, ammonium, pH, phosphate, and sulphide levels. In this regard, the strong importance of pH explaining the *Archaea* variation (44%; Fig. 4b) supports the hypothesis that Sacca di Goro surficial sediments can be dominated by pH-sensitive archaeal functional groups, such as ammonium oxidisers.

## Conclusion

The environmental parameters confirmed that Sacca di Goro lagoon is characterised by physico-chemical (i.e. pH and salinity) and trophic (i.e. CHLA and BPC) gradients with pronounced seasonality. We investigated the archaeal and bacterial standing stock and were able to associate changes according to certain environmental parameters. Bacterial and archaeal abundance are differentially controlled by environmental factors, suggesting that they might have a different role in aquatic biogeochemical processes. One of our main findings was that *Archaea* are a numerically important component of the benthic microbial assemblages in the lagoon, especially at sediment layers below a few centimetres. Also during winter, a season of decreased temperature, *Archaea* remain stable in abundance. We conclude that *Archaea* may play neglected role in C cycles in Mediterranean coastal lagoons, which should be elucidated in future studies for a better understanding and management of lagoon ecosystem biogeochemistry.

## Electronic supplementary material

Below is the link to the electronic supplementary material.

Supplementary material 1 (PDF 359 KB)
