# Supplementary material for: Environmental Drivers Controlling Bacterial and Archaeal Abundance in the Sediments of a Mediterranean Lagoon Ecosystem
Source: Curr Microbiol. 2018 May 15;75(9):1147–55. doi: 10.1007/s00284-018-1503-3 (PMC6096605; doi:10.1007/s00284-018-1503-3)
Supplement: Supplementary file 1 — Supplementary material 1 (PDF 359 KB) [file 284_2018_1503_MOESM1_ESM.pdf]

Current Microbiology

Supplementary Materials for

Environmental drivers controlling bacterial and archaeal abundance in the sediments of a Mediterranean lagoon ecosystem

Claudia Pala[1,2,3], Massimiliano Molari[2,3], Daniele Nizzoli[1], Marco Bartoli[1], Pierluigi Viaroli[1], Elena Manini[2]

[1] Department of Chemistry, Life Sciences and Environmental Sustainability University of Parma, Parma, Italy
[2] Institute for Marine Science - ISMAR, National Research Council of Italy – CNR, Ancona, Italy
[3] Max Planck Institute for Marine Microbiology, Bremen, Germany

Corresponding author:
Claudia Pala
Address:
Max Planck Institute for Marine Microbiology, Bremen, Germany
Celsiusstrasse 1, 28359
Bremen,
Germany
E-mail: cpala@mpi-bremen.de

The PDF file includes:
-Supplementary Material and Methods to implement FISH and total microbial cell numbers protocol.
-Figure S1 Organic matter content in terms of Biopolymeric Carbon, Proteins, Lipids, Carbohydrates and Chlorophyll-a at Giralda, Gorino and Mare during summer 2011 and winter 2012.
-Figure S2 Principal component analysis of environmental variables at Giralda, Gorino and Mare in the surface sediment layer (0-0.5 cm) and **b)** Principal component analysis of organic matter composition and chlorophyll *a* at Giralda, Gorino and Mare in the three sediment layers during summer 2011 and winter 2012.
- Table S1 Output of multifactorial ANOVA: the differences in the composition of the *Bacteria* and *Archaea* have been tested between the different layers of sediment, different stations and different sampling periods.

Supplementary Material and Methods

FISH and total microbial cell numbers protocol.

The FISH technique allows the visualization, identification, and enumeration of single cells. Through the hybridization procedure ribosomes (rRNA) within a cell are stained by a fluorescent oligonucleotide probe - in our case specific for *Bacteria* and *Archaea*. The procedure involves the following steps: (i) sample fixation, (ii) cell extraction, (iii) sample filtration, (iv) hybridization, (v) washing to remove the excess of probe, (vi) mounting on slides for viewing and enumerating under the epifluorescence microscope. The samples were fixed with 2% formaldehyde (final concentration) for 24 hours at 4°C, followed by three time washing with PBS (145 mM NaCl, 1.4 mM NaH_2PO_4, 8 mM Na_2HPO_4, pH 7.4) and centrifugation (2500 rpm for 5 minutes). Finally, the cells were resuspended in 5 ml PBS and 96 % ethanol (1:1). The cells were separated from sediment particles by sonication (three cycles of 3 minutes each with 30 seconds intervals with shaking).

The volume of 250 µl were added to 5 ml of PBS and filtered onto polycarbonate filters with 0.22 µm pore size. The filters were immersed in 0.2% (w/v) Low-Gelling-Point Agarose in reagent-grade water (0.2 µm-filtered) at 35°C in a non-humid environment for 20 minutes, then dehydrated in increasing concentration of ethanol (50%, 80%, and 96%) for 1 minute each at room temperature. The hybridization of the samples was achieved by adding to each filter a 10:1 mixture of hybridization buffer (900 mM NaCl, 20 mM Tris-HCl, pH 7.4, 0.01% SDS, 35% formamide) and probe labelled at the 5'-end with the fluorescent molecule Cy3 (50 ng/μl^{-1} final concentration).

The hybridization was carried out in the dark in a thermostatic room at a temperature of 46°C for 2.5 hours. The samples were then washed in washing buffer (20 mM Tris-HCl, pH 7.4, 900 mM NaCl, 0.01% SDS, 5 mM EDTA) to

remove excess and unbound probe. This operation was performed in the dark in a thermostatic room at 48°C for 15 min. The filters were then rinsed in reagent grade water and left to dry.
After the washing, the filters were stained with 30 μl of 4',6-diamidino-2-phenylindole hydrochloride (DAPI; 1 μg ml^{-1}) and incubated in the dark for 3 minutes. Subsequently the filters were washed in 96% ethanol for a few seconds, rinsed with reagent grade water, and let air dry. Finally, the samples were mounted on a slide with 20 μl of a solution of PBS-glycerol-ascorbic acid (anti-fade).

Fig.S1 Organic matter content (BPC, PRT, LIP, CHO, CHLA) at Giralda, Gorino and Mare during summer 2011 and winter 2012.

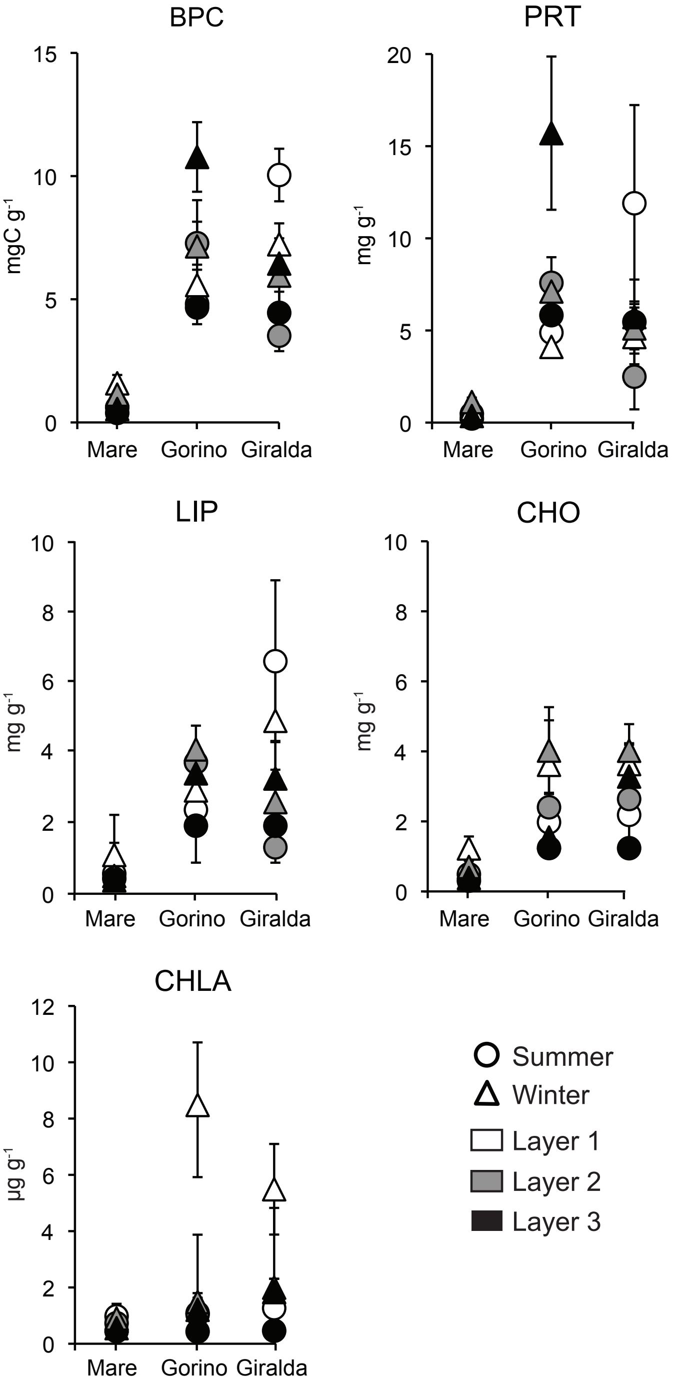

Fig.S2

a) Principal component analysis of environmental variables at Giralda, Gorino and Mare in the surface sediment layer (0-0.5 cm) and **b)** Principal component analysis of organic matter composition and chlorophyll *a* at Giralda, Gorino and Mare in the three sediment layers during summer 2011 and winter 2012.

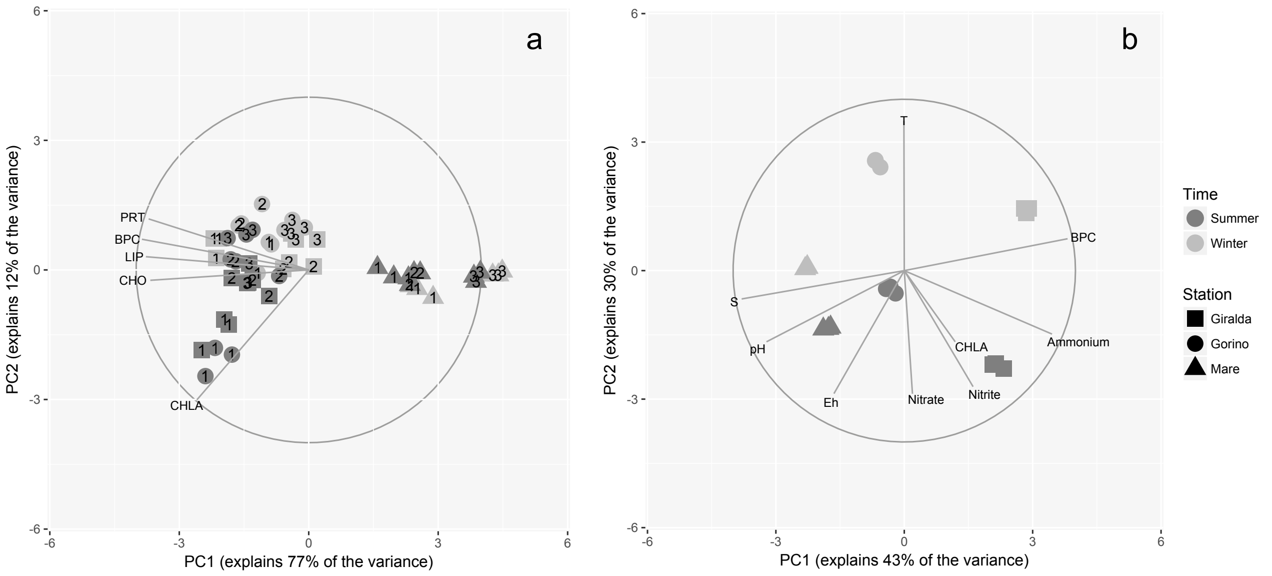

Table S1 Output of multifactorial ANOVA: the differences in the composition of the *Bacteria* and *Archaea* have been tested between the different layers of sediment, different stations and different sampling periods.

	Df	Sum Sq	Mean Sq	F value	Pr(>F)	Redundance
**Bacteria**						
Time	1	8.551	8.551	43.972	3.98E-08	**36%**
Site	2	2.156	1.078	5.544	7.12E-03	9%
Site:Layer	3	4.383	1.461	7.513	3.64E-04	19%
Residuals	44	8.557	0.194			
Total		23.647				
**Archaea**						
	Df	Sum Sq	Mean Sq	F value	Pr(>F)	Redundance
Time	1	0.212	0.21199	6.39	1.51E-02	9%
Site	2	0.1273	0.06366	1.919	1.59E-01	6%
Site:Layer	3	0.4425	0.1475	4.446	8.18E-03	**20%**
Residuals	44	1.4598	0.03318			
Total		2.2416				

Df: degrees of freedom; Sum Sq: sum of squares; Mean Sq: mean square; F: statistic *F*; Pr (>F): probability level.
